# Understanding long-term sick leave in female white-collar workers with burnout and stress-related diagnoses: a qualitative study

**DOI:** 10.1186/1471-2458-10-210

**Published:** 2010-04-26

**Authors:** Hélène Sandmark, Monica Renstig

**Affiliations:** 1 School of Health and Medical Sciences, Public Health Science, Örebro University, Sweden; 2 Department of Medical Sciences, Occupational and Environmental Medicine, Uppsala University, Sweden; 3 Women's Business Research Institute, Stockholm, Sweden

## Abstract

**Background:**

Sick leave rates in Sweden have been significant since the end of the 1990s. In this paper we focus on individual female white-collar workers and explore various factors and setting-based sources of ill health in working life and in private life, in order to understand impaired work ability, leading ultimately to long-term sick leave.

**Methods:**

A qualitative methodology was chosen, and thematic, open-ended interviews were carried out with 16 women. The interviewees were strategically selected from a cohort of 300 women in full-time white-collar jobs in high-level positions, living in three urban areas in Sweden, and on long-term sick leave ≥90 days. A qualitative content analysis was carried out.

**Results:**

The informants in the study were generally well educated, but a few had surprisingly little formal education considering their salary level and position on the labour market. The women were in professional positions more commonly held by men, either as specialists with some degree of managerial role or as executives with managerial responsibilities. Both external and internal stressors were identified. The analysis indicated that being in these gender-typed jobs could have induced sex discrimination and role conflicts. The women expressed strong agreement regarding success in working life, but emphasised the lack of competence matching in their present jobs. They also lacked the sense of having a rewarding job, saw leadership as weak, and disliked their present workplace and colleagues. Impaired health may have hindered them from changing jobs; conversely, their locked-in positions could have resulted in deterioration in their health status. The women displayed personal overcommitment, both at work and in private life, and had difficulties in setting limits.

**Conclusions:**

Factors in working life, as well as in private life, played an important role in the informants' deteriorated health and long-term sick leave. Job and workplace mismatching, problems in connection with company profitability, and poor leadership contributed to stress-related symptoms, resulting in reduced working capacity. On the basis of these findings, attention should be paid to identification of early indicators of exhaustion, and measures should be taken in work settings and in working life as a whole to promote retained work ability.

## Background

The social status and economic behaviour of women in the industrialised part of the world have changed, and their participation in working life has increased, Not only do they have a high rate of participation in the workforce; they have also made advances in management, which used to be a male domain [1]. However, there is still a gender gap in managerial posts in OECD countries, with a higher proportion of men than women working as managers or directors. Thus management positions are still divided by gender, and women are in the minority [2,3]. This means that women in managerial positions mostly have male colleagues, and that management teams are male-dominated. This situation does not correspond with the currents rates of higher education, in preparation for a traditional career, as levels of female attainment in tertiary education in the OECD area, including Sweden, exceed those of men [4].

A high rate of sick leave is the public health issue that has dominated occupational life in several European countries, including Sweden, during the past decade [5,6]. In an international perspective the sick leave rates in Sweden have been significant, and at the end of the 1990s there was a considerable increase in long-term sick leave in the Swedish labour force. Women in the Swedish population have taken twice as much sick leave as men [5,6]. However, towards the end of this decade there has been a slight decrease in long-term sickness absence and early retirement [6]. For white-collar workers in Sweden, the increase in long-term sickness absence between 1998 and 2002 was 230%, and for white-collar women with the highest wages the increase was 335% during the same period [7].

Over the past decade, studies have shown a deterioration in the psychosocial work environment and along with it, a significant rise in sickness absence in Sweden [8]. This has been more pronounced for the female workforce, which makes it a gender issue, characteristic of the gender-segregated labour market in Swedish working life [9]. Downsizing in the public sector, as well as in private companies, has placed extra pressure on co-workers. There has also been organisational restructuring of companies and globalisation of enterprises during this period, which has changed the prerequisites for the individual. All these changes, along with the increase in female participation in working life, probably affect health and well-being among women in the labour force [10-12].

Sickness absence is not entirely identical with biomedical dysfunction; it is more complex and also includes psychological as well as social dimensions. Effective prevention of ill health and reduction of sick leave require knowledge and understanding of target populations, such as a knowledge of functioning at the workplace, demographics, and the psychosocial situation.

The starting point of this study is that sickness absence mirrors the balance of a person's resources in relation to demands from work, family and societal life, rather than solely biomedical disease [13]. It has not been investigated to any great extent how this is connected with the occupational groups studied here. The focus of this paper is on individual female white-collar workers, and the aim is to explore various factors and setting-based sources of ill health at work and in private life, in order to gain an increased understanding and a broad picture of impaired work ability, leading ultimately to long-term sick leave.

## Methods

An interview study was chosen in order to explore and describe the everyday experience of female white-collar workers in their occupational and private life, prior to their period of sick leave. Thematic and open-ended interviews allowed the informants to express their experience, instead of being guided by the researchers' a priori ideas of relevance [14]. The approach in this study was thus based on qualitative research methodology, as the intention was to gain an understanding of actions and meanings in the women's lived experiences. In the face-to-face meeting with the informant, the researcher therefore endeavoured to see things from the subject's perspective, but the focus in this study was on content and not on interaction [15,16].

### The Swedish social insurance system

The social insurance system in Sweden is uniform throughout the country, funded by the Swedish state, and administrated by the Swedish Social Insurance Agency. Everyone who lives or works in Sweden is covered by social insurance. Sickness compensation is possible in the case of a reduction in work capacity caused by sickness or injury. The insurance distinguishes between personal sickness absence and staying at home to take care of sick children, which is covered by a special insurance within the Social Insurance Agency [17]. For employees in the private sector in Sweden, a mutual insurance company, Alecta, operated at the time of this investigation, on behalf of the Confederation of Swedish Enterprise and the Federation of Salaried Employees in Industry and Services, to provide additional compensation in the case of long-term sickness lasting more than 90 days [18].

### Setting and interviewees

The respondents in this study were strategically selected from Alecta's registers of female white-collar workers in high-level positions, living in three urban areas in Sweden (Stockholm, Göteborg and Malmö areas) and on long-term sick leave due to stress-related diagnoses, burnout or fatigue syndromes or minor depressions. The register at Alecta has a total of 1,170,000 policy-holders, and we included women from a cohort of 300, who had been continuously sick-listed for more than 90 days, between February 2004 and October 2004. Further, the sampling process included the following criteria: having a full-time position with a monthly salary of at least SEK 30,000 (EUR 2,900) to ensure that the women were in high-level positions; being of different ages within the pre-determined age group of 31 to 52 years of age; and representing all three chosen regions. The first and second author made the selection. All women invited to the interviews agreed to participate. Their personal characteristics are shown in Table 1.

**Table 1:** Personal characteristics of the interviewed women

Age	Children	Marital status
31	0	Single
31	1	Married
33	0	Divorced
34	0	Living with a partner
34	0	Living with a partner
35	2*	Married**
37	2	Married
41	0	Married
42	2	Married
44	2	Married
47	3	Living with a partner
47	0	Divorced
48	1	Living with a partner
50	2	Living with partner**
50	1	Married
52	0	Living with a partner

* plus husband's 3 children

** previously divorced from her first husband

### Interviews

Thematic, open-ended interviews were performed by four women: a public health researcher (first author), an experienced careers counsellor (second author), a graduate economist, and a graduate social worker. An interview guide was drawn up, including themes considered relevant to the research question. The interview guide included the following themes: occupational history and present work, family and social network, health and sickness absence. However, in order to avoid any preconceived ideas, it did not include any pre-constructed questions. Accordingly, the interviews were unstructured; i.e., all areas were covered in all interviews, but the sequences in which they appeared differed [19]. The open-ended interview approach meant that the interviewer asked follow-up questions related to the interviewee's answers. Each interview lasted about one hour. Sixteen interviews were carried out in 2004. The interviewees' work positions are shown in Table 2.

**Table 2:** The informants' highest education and qualifications, previous working positions, and work situation at the time of the sick leave

Highest education/qualifications	Previous work	Work situation at the time of the sick leave
University Bachelor of Science	Marketing, marketing manager, managerial responsibility	Became unemployed during sickness absence Regained employment following legal action, but not interested in returning to this work position
Upper secondary school, supplemented with individual courses	Purchasing assistant, purchasing manager, managerial responsibility	Purchasing manager, but given redundancy notice
University Lawyer	Business area manager Bank lawyer, financial advisor	Business area manager at training firm Managerial role shared with male co-worker
University Computer scientist	IT consultant	Computer scientist with responsibility for 3-4 people Company recently sold to a US-based firm
University Human resources specialist	HR administrator, managerial responsibility	Personnel manager for part of a US management company
University Behavioural scientist	Consultant	Employed at an engineering company
9-year compulsory education, supplemented with individual upper secondary courses	Business economics	Group manager for 35 people, later four people, and then project manager
Technical upper secondary school, supplemented with courses in marketing and market economy education	Sales, sales manager	Marketing manager at an office equipment company
University Humanities	Designer	Designer/specialist
Technical university Civil engineer	Research, computer specialist	Development of information systems and computer software
Upper secondary school IHM	Information, marketing	Manager at information department
University Media programme	Media sector	Editor-in-chef
University Bachelor of Science	Secretary, controller	Financial manager
University No degree but courses in marketing and economics	Marketing assistant, advertising manager, marketing manager	Marketing manager at a major international company
Technical upper secondary school Engineer, short courses, IHM	Engineering sector, managerial role	Managerial responsibility for about 30 people in an engineering team
University Lawyer	Healthcare, economics, computers, consultancy manager	Regional manager at a training company with personnel responsibility for 60 people

### Data analysis

The interviews were recorded and transcribed. The transcriptions were read through several times independently by the authors, so that each could obtain a general impression and become familiar with the data. Thus, the transcribed texts were first subject to independent analysis. After this, the data were reduced and categorised through an interactive process of discussing and revising to reach consensus by all four interviewers. The approach was inductive in order to identify new insights [19]. The words, sentences and paragraphs, i.e. the meaning units, representing anything that could be connected to the aim of the study, were identified according to principles which have been described by Tesch [20] and other researchers [21] as de-contextualisation. The meaning units were condensed and put together, and then organised into categories constituting the descriptive level of the content, i.e. the re-contextualisation phase.

Themes were created by linking the categories demonstrating the manifest content of the interviews [22,23]. The themes were discussed by the two main authors to gain confirmability. The themes were compared with the whole body of interviews in order to validate them to their original context. The tentative themes were also discussed and then revised. As the analysis focuses on the manifest content, it has to some extent been possible, as described by Krippendorff (2004), to indicate some quantification of the informants' descriptions [23]. The quantifications were intended to indicate the direction of the descriptions.

### Ethical considerations

The prospective interviewees received an introduction letter, inviting them to participate and informing them about the aim and confidentiality of the study, and how it was going to be conducted. Informed consent was obtained, and also once again immediately before the interview started. The Ethical Review Board at Karolinska Institutet in Stockholm approved this study.

## Results

The analyses resulted in three themes and nine categories, providing a picture of the sick-listed respondents' earlier working life situation and experience at work, as well as their health and private life situation at present and at the time just before their sick leave. Three categories are related to the theme "conditions in working life", which concerns the reported situation at work immediately before sickness absence. The theme "experience of working life" covers the women's reported experience at work, including workplace culture, relations to colleagues, management and basic company values. The third theme concerns the respondents' reported health status and stress responses (Table 3).

**Table 3:** Themes and categories emerging from analysis of the interviews

Theme	Category
Conditions in working life	The men's world
	Restructuring and profitability problems
	Poor leadership
Experience of working life	Career focus, but competence mismatching
	Wrong workplace and colleagues
	Hard to set limits
Deteriorated health and imbalance	Incidents in private life
	Imbalance in work- and private life
	Stress-related symptoms and too little recuperation

### Conditions in working life

#### The men's world

"I've always been on my own - it was like a gentleman's club at the company. Very few women managers. I've often had to accomplish more, and when I suggested something new, it was always questioned because I'm a woman."

Being the only woman among men in the management teams was hard and often challenging. The respondents did not know the male work culture and game rules well enough, and expressed a lack of mentorship and role models.

One of the interviewed women received a top management position in the human resources department in a major company just before she was thirty years old. Although she had good support from the administrative system she had little support from her superiors and senior managers. She had not had the benefit of coaching or a mentor, and was simply encouraged to take more and more responsibility and to work harder. Finally she exceeded her limits, her health deteriorated, and she became exhausted. Her diagnosis when she was sick-listed was fatigue syndrome.

"I haven't had any female role models and mentors in working life."

One of the women said that she had never been absent due to sick children until one day when her son was really ill. Among her colleagues, who were all male, it was not legitimate to take time for family matters. She was the only women in the management team where she worked, and all the other women at the workplace were in administrative and non-executive positions. She stayed at home for one day to be with her son, and after that her boss asked her if she had lost focus on her occupational work. Later when she herself was on sick leave her boss did not get in touch with her, and there was no human resource department at the company. Another respondent reported that the family situation of her male colleagues was usually different compared with hers. She explained that the wives of men in the management team often worked part-time and took responsibility for children and household work, and stayed at home when the children were ill to let the man concentrate on his paid work and career. She stressed that the reverse rarely existed regarding career women and their partners.

"The men always have a wife."

The informants had experienced that they were increasingly expected to put up with more, simply because they were women. They reported that there were more equal opportunities when times were good, but in harder times, when there was downsizing at the workplace, there was often a backlash and regression in this area.

### Restructuring and profitability problems

The tougher labour market climate in recent years has resulted in greater competition, profitability problems, restructuring, sell-offs, and austerity measures at companies. This has increased pressure on women and their performance at work. The informants had taken part in staff lay-offs either in their positions in middle management or because they were employed at the company's personnel department. Some of the women had previously been made redundant as part of cutbacks and reorganisation. Poor profitability in operations had been a problem. This had not only led to reorganisations and staff cuts, but also frequent management changes.

### Poor leadership

The Swedish democratic management style is less evident in companies when international executives enter the Swedish labour market, or when companies that have previously been Swedish are acquired by foreign companies. One of the informants talked about the time just before she began her sick leave. Her career had been successful and she had gradually been given new and higher positions in her working life. Things were fine until she began to work for a major international group. During a relatively short period of time she had had different managers from various European countries: from Sweden, Germany, the UK, Belgium, and Scotland. She experienced the foreign managers as more authoritarian and old-fashioned in their management styles, and felt unfamiliar with the management culture that these managers introduced.

"Each was worse than the other. You can still have discussions with managers in Sweden, but this is not the case in Germany."

Just before her sick leave, one woman had worked many hours. Her manager thought nothing of phoning her on a Friday evening and demanding that she had to complete a series of tasks by Monday morning. Since her sick leave she had reflected on this and now understood that she had made too few demands when this happened.

Several of the informants had had management positions, despite the fact that they were not really interested in this, often because there were too few candidates; or they shared a managerial role with a man, and were then responsible for personnel issues. The managerial role often meant difficulties and problems for these women.

"It started 2-3 years ago when I became co-manager with a male colleague who was a very poor leader. I was to compensate for him. A weak leader who needed support. I didn't think it was going to be such a problem."

### Experience of working life

#### Career focus, but competence mismatching

"I loved sitting in a black suit together with a load of Americans at executive meetings and travelling first class."

Respondents talked of a strong focus on their work and on working life, and a clear professional identity. Before their sick leave and before the problems with their health and well-being, they had had a high level of satisfaction with their profession. They had spent significant amounts of time and energy on their work, and had sought and accepted many challenges. They had been in demand on the labour market and had usually not applied for jobs but had been offered various posts. The informants were at different stages in their career and they were of different ages.

"I have never applied for work. I have been employed at typically male workplaces - I've been lucky with good bosses and have had a good deal of freedom to develop myself and my employees, though it was important to deliver."

Early in their professional lives, only a short while after completing formal education, the youngest informants had received an independent job with substantial demands and expectations from employers, along with a good salary and other benefits. However, they had not always been able to cope with highly qualified and demanding jobs at an early stage in their career and lacked working experience. One of the younger informants was given what she called a dream job as her first employment before the age of 30. As personnel manager, she worked 80 hours per week and every weekend, and was part of setting up a business operation. Her tasks included firing personnel and this became a large part of her job. Eventually she found her work was neither interesting nor stimulating any longer. She was never at home; "she toiled and cried".

A belief that a long period of employment would reduce the risk of losing their jobs during cutbacks had convinced the informants not to leave an unsuitable work situation or workplace. A woman who had been at the same workplace for more than 20 years had understood that she should change employer to find a profession that suited her better. She had experienced difficulties keeping up with the rapid developments within the field of IT; her skills were now out of date, the workplace was turbulent and the work demanding. However, she dared not become the last person to be employed at a new workplace, as this would mean she would be the first to lose her job if business became slack and there were staff cutbacks. The woman had children in school and her husband had recently been made redundant, so she felt that she must continue as she had the main responsibility for providing for the family and wanted a relatively secure income.

Some informants found themselves in a position where their skills were no longer required. One woman worked at a company which had recently undergone a major reorganisation. Her managers had been unclear in their communication with her about her work and position in the organisation. Previously she had always felt appreciated at her workplace, but this had changed. Just before she took sick leave she experienced some problems with her managerial role. The woman in question had little formal education in relation to her role at the company and her earlier career.

Another woman wanted more work, but she felt the company had a nonchalant attitude to her. She was never called to meetings and felt that she no longer had any real function at work. She had applied for other jobs, but had not been offered any other work and believed this was linked to the fact that she did not have any formal education apart from 9-year compulsory education. Her colleagues avoided talking to her about the current situation. Her immediate manager did not discuss the situation with her but talked to her colleagues about this when she was not present.

### Wrong workplace and colleagues

Being at a workplace that is not suitable does not necessarily mean having the wrong job or profession. There is however no clear dividing line between being "locked in" at workplaces and at a certain work, and it can sometimes be difficult to know what should be changed.

Informants told of colleagues and workplaces that did not match their previous experience and direction. One of the respondents had been active for some time at a workplace which specialised in research and development. The training company she now worked at did not suit her and there were constant misunderstandings and tense situations. She had a sense of being bullied and that information about work issues was withheld from her. Project work was run differently compared with her earlier work experience.

One informant worked as part of an interdisciplinary team where age, gender, and professional profile separated her from her colleagues. The values and workplace culture prevalent within the working group were not the same as hers.

"I feel that it's actually the job that's not right for me. But I've always felt like a loser, that I failed every day. Alone among all the techniciens. All the 50-year-old men were really vicious in their attacks. I'm extremely lonely."

### Hard to set limits

"During my sickness absence I've reflected on the fact that for some time I've had difficulties setting limits. It would probably have been easier if I'd been a man, but I've felt pressure on me to achieve more because I'm a woman."

Since childhood the women had felt pressure from their parents or close friends, or made demands on themselves to be high-achievers in everything they did. They had found it difficult to say no to work for which they lacked skills and experience, or to work that was too extensive and that really required several people to carry out. They had found it difficult to complete some of the work and had therefore been obliged to spend many hours on it. This had created frustration, particularly when in a longer perspective it had impacted their health and private life.

Because these women had wanted a challenge and been prepared to invest time and energy in their occupation, they had found it difficult to reject the demands made by this work. In the posts they currently held or had held they were expected to spend significant amounts of time at work, but also to have a clear awareness of priorities. The women apparently lacked this latter quality and boundaries became blurred; they had had difficulty saying no and setting limits, which also meant they often completed tasks for co-workers who had not had time or had not done the tasks correctly. It was discernable in the interviews that there had been problems in delegating.

### Deteriorated health and imbalance

Some of the informants were living in relationships with a partner, some were single, and more than half of them had children (Table 2). Some were in a life-long couple relationship, while others were living in a new or relatively new second relationship. The interviews provided no evidence of any major problems or conflicts with current partners; rather that they were supportive during the women's careers, and during the current period of sickness absence. Children were described as good and conscientious.

"The children are no problem, they are active and doing well in school."

### Incidents in private life

Most of the respondents had had traumatic experiences in their childhood years which had had an impact on them. Early in life, due to parents' addiction or early death they had had to take responsibility both for themselves and for others, such as younger siblings.

In adult life, demands from work had been linked to events and problems in the private sphere. The respondents told of the loss of a newborn child, the death of several relatives within a short space of time, unsuccessful adoption processes, and difficult child custody disputes. The simultaneous exposure to stress in both working and private life had resulted in deterioration in health and a stress collapse with subsequent long-term sickness absence.

### Imbalance in work- and private life

"I have always thought I had endless amounts of energy. I feel work has encouraged us to work a lot, no one sets limits, not even society. Then children need to be taken to different activities - it's difficult to get out of it."

The informants with children related that their ambition had been to cope with both work and children in a good way. However, the position of women in working life had not allowed them to stay home from work to take care of children when they were sick or needed care. Despite this, the informants had taken the main responsibility for a demanding career and for their children, home and household. The planning they had undertaken had been an attempt to combine employment with responsibility for home and family. From an early age they had felt demands from their parents, other relatives and friends or taken responsibility themselves, always to be good and high-achievers in everything they did. One woman said she lived by the motto:

"It should not be noticeable at home that I have a demanding job that requires a lot of my time. And it should not be noticeable at work that I am responsible for a home and a child."

One woman said she believed her sick leave was a result of the fact that she had had too much to do at work. However, she had always found it stimulating to have a career, but did not want her child to suffer for this. She never watched TV or went to the cinema. Instead, she took care of her child, did the washing, cleaning and everything else in the home, in addition to her work.

"I was so aware of my deadlines, but now I understand that private life is also important. I'm bad at saying "no", I couldn't bring myself to say that I hadn't had time..."

### Stress-related symptoms and too little recuperation

"I was asked to redo the entire budget...an entirely new budget, I worked all weekend. On Monday morning I couldn't get out of bed, I felt incredibly tired, as if I'd been awake for a week, pressure on my chest, heart pains, breathing problems. Without being aware of it, I had stopped eating, had lost 7-8 kilos since October, I was never hungry, had eaten enough after 2-3 mouthfuls. I went to the doctor, was put on sick leave for 10 days at first, had serious spasms."

During the period just before the sick leave, respondents had become increasingly tired and experienced difficulties in handling exposure to external and internal stress. Both external demands made by people around them and internal demands made by the women themselves had become too much. The long-term stress increase had manifested itself as extreme overexertion.

Symptoms from the negative stress appeared gradually and the women related initial symptoms of tiredness, sleeping problems, a gradual increase in depression, and dissatisfaction with work. One woman described an "everyday life with no fun". This gradual deterioration included irregular eating habits and loss of control over feelings of hunger and the intake of nutrients. In some cases isolated anxiety attacks and an increasing aggressiveness were noted. The long-term and high stress increase finally resulted in a more or less acute collapse.

"I was hyperactive and tired, extremely stressed. The last job just broke me. I passed out at my desk"

The women had little time for themselves, or for rest and recuperation. They did not consider their own health and neglected their early symptoms. During their sickness period they reflected on this lack of focus on their own health and the necessity for recuperation. Although not all of the respondents had children, the whole group stated that they did not get much recuperation due to long working hours and a strong commitment to their gainful work.

"Own time, what's that? You have to steal time for yourself."

The informants had few social contacts outside their workplace and home, as they had worked to such an extent that it was difficult or almost impossible to build up or maintain a social network due to lack of time. This was also the case for the women without children. As health deteriorated and a long period of sick leave began, this lack of a social network and friends had been further accentuated when the respondents no longer met their colleagues.

One of the younger women without children said that because she had worked so much she had not had time to establish a circle of friends. Instead she socialised with her partner's friends. Now that this relationship had ended she did not have many friends left, but she understood the importance of the friends she still had.

Experience varied regarding contacts with public healthcare services, occupational health services, and the Social Insurance Agency at the outset of sickness benefit and later rehabilitation. A few respondents were very satisfied and felt they received the correct treatment and good care. Others were disappointed and felt there were shortcomings in treatment, service and accessibility. They found it difficult to get through to and book appointments at welfare centres, or they had to travel long distances to a care centre. The first contact with the healthcare services had sometimes not resulted in any help or the wrong help, although at a later stage there were fewer problems when a doctor or therapist provided the right treatment or therapy. This had added stress and discomfort to the original problems. Help outside the regular healthcare system helped to alleviate symptoms, such as regular talks with older colleagues who acted as mentors.

The occupational health services had generally offered good support and the informants felt that they had received understanding for their tough work situation and symptoms of ill health. The occupational health staff were regarded as being aware of the correct action to take for the problems and able to offer the right support and treatment. However, contacts with the Social Insurance Agency were seen as very negative. Some had been questioned about documents and reports that were missing in their files. None of the respondents had received any support from the Insurance Office during the sickness benefit process.

## Discussion

The objective of this study was to gain understanding regarding impaired work ability, i.e on long-term sickness absence, in the context of female white-collar workers in high-level positions in Sweden. Work ability or inability is a complex issue and there are many layers of influence, such as internal factors connected to skill and context, and to interactions in the societal and organisational culture [24-27]. This complexity has been confirmed in this qualitative interview study.

In this section the results will be discussed in relation to long-term sickness absence, and the findings will be compared with earlier research in related fields and connected to theoretical models and concepts used in work health research. Methodological considerations, trustworthiness, and transferability of the findings will also be discussed in this section.

The women in the study were generally well educated and most had a final degree from a university or institute of higher education. A few had surprisingly little formal education considering their salary level and position in working life (Table 2). The informants' education and profession is not within the framework for the gender-segregated labour market which is characteristic of Swedish working life [4,28,29]. The women have posts that are more commonly held by men and are not horizontally segregated, i.e. in the typically women-dominated sectors and jobs on the labour market. Nor were they vertically subordinate, i.e. in jobs at the bottom of the hierarchy at workplaces. The informants were on the contrary in leading positions, either as specialists with some degree of managerial responsibility or with managerial responsibility as managers in a linear organisation.

The working environments where the women are active are male-dominated. The women indicated that this could have induced sex discrimination, role conflicts, and misunderstandings, which seems to have played an important role in their deteriorated health. Similar findings have been reported in studies since the 1970s [30,31]. According to gender role theory, a stronger family involvement is more congruent with female gender and, accordingly, women in management positions could challenge traditional gender roles [32], which might entail perceptions about values and relations in the management teams or in relations between male and female managers.

Our findings are consistent with results from studies stating that competent women in male gender-typed positions can be disliked, not encouraged, and have a hard time building relations with their male colleagues, primarily because of their gender [33,34]. Due to this, their performance in male gender-typed tasks can be devalued and their competence denied [35].

One feature in the informants' descriptions is duality and ambiguity, implying both negative and positive dimensions. This is apparent in the categories directly related to the women's positions in working life: "Career focus, but competence mismatching", and "Wrong workplace and colleagues". The women expressed strong agreement on succeeding in working life, but stated that there had been no competence matching in their present jobs. Despite this double-edged stance, the informants seem not to have given up on finding the right position and continuing a professional career. In some cases impaired health probably stopped a job change, which could have helped to save strength. On the other hand, their "locked in" positions could have had a detrimental impact on their health status. The informants' impaired health could also be a simple reason for not changing jobs due to "last in, first out" rules on the Swedish labour market and a striving not to compromise their present work positions. Our findings are in concordance with the results in a recent study [36] of almost 6,000 men and women in Sweden, where it was shown that "locked in" positions in occupation or workplace are associated with long-term sick leave.

As the women's gainful work is reported as not entirely rewarding, this could be considered a threat to work ability and well-being, addressing studies which claim that it is important to maintain a mutual balance between efforts needed for performing a job and the reward obtained [37,38]. Not being rewarded for one's efforts or being unjustly treated have been found to be important issues in the process leading to burnout and emotional exhaustion in working life [39]. If high work-related demands recurrently occur in combination with low rewards, it is possible that this can cause stress-related disorders and sustained strain reactions, with adverse effects on both physical and mental health [37-39]. The effort-reward imbalance model of work stress is applicable if people are exposed to heavy competition or if they are intrinsically motivated to engage in excessive work-related commitment. The informants probably overestimated their own coping resources and were not fully aware of their own contribution to the non-reciprocal exchange. Their overcommitment had been extensive and also included considerable efforts and responsibilities within the private sphere. The women had used a great deal of their resources both in family- and work roles, having had far too little recuperation to maintain their health and well-being. This can be considered a coping style, which is a psychological construct and presented as performance-based self-esteem. Self-esteem is a concept based on accomplishments that is used in burnout modelling [39]. This is a concept, or rather an important personal trait, in high psychic distress and strain, which can lead to a failure in the balance between resources and perceived strain. It has been concluded that under certain circumstances this pattern can predict burnout and maladaptive stress reactions [40], which agrees with our results. Depite their strong focus on their work performance and career, it is certainly possible that the women reduced their professional efficacy due to their overcommitment and response to stressors.

Our findings are connected with the well-known job-demand-control-support model, which in several studies has been applied by showing that imbalance between psychosocial demands, decision latitude and social support, impacts health functioning and could lead to mental dysfunction [40-43]. Job demands were reported as high, and the results indicate that decision latitude was limited, despite the fact that the informants were in management positions. Their coping style could have hindered them in achieving a balance between work demands and decision latitude and thus a high degree of job control, showing that the imbalance might to a great extent be self-imposed. Compared with jobs that women usually have on the gender-divided Swedish labour market, the informants in this study had positions in working life that are not usually subordinated, but generally have a high decision latitude and opportunities to control and balance work. In earlier research, the high demand component at work has been found to predict emotional exhaustion [44,45]. Low levels of work demands, control, and support in British civil service employees have been found to be associated with higher rates of short and long spells of absence in men, but to a lesser extent in women [46]. However, results from a cross-sectional Swedish population study on long-term sick leave showed the highest associations with women in high-strain jobs in the private sector, which corresponds well with our results [47].

The failing support found in this study concerned immediate supervisors, co-workers and the organisation or workplace at large. The impact of failing social support on sickness absence from colleagues has been found in earlier studies. In a sample of the Swedish working population it was found that for long spells of sick leave, poor support from colleagues was related to long-term sick leave, and that good support from superiors, but not from colleagues did not change these factors [48]. In a Finnish study of employees in the private sector, lack of support from co-workers increased the frequency of long sickness absence among men, but not among women [49].

The results from our study indicate that there was a connection between the reported restructuring and profitability problems in the women's companies and the leadership styles suggesting a lack of support from managers. Economic problems could certainly entail negative stress for the management; this in turn could contribute to a poor working climate, resulting in negative health consequences for the employees, and causing or contributing to long-term or frequent periods of sick leave [50]. It has also been found that leadership which is based on exerting authority and not oriented towards solving problems through discussions is related to a higher level of sickness absence [51], while inspiring and communicative leadership, suggesting a transformational leadership style, has been shown to be related to lower rates of sickness absence [52].

### Methodological considerations

The trustworthiness of a qualitative interview study depends to a large extent on the interaction between researchers and informants, and on the analysis of the data [19,53]. A qualitative method was chosen for data collection in this study, with an explorative and inductive approach in analysies, in order to obtain a wide picture of the women's experience of work inability in the context of women in high-level white-collar positions in the private sector on the Swedish labour market. The qualitative method also allowed flexibility, and made it possible to study interrelations rather than to spot causes.

The interview situations were planned according to the wishes of the interviewees in order to create an unstrained setting. The outlined interview guide allowed an inductive strategy to derive the predominant themes reflected in the interview transcripts. The interview guide did not hinder this strategy, as it was very short. After sixteen interviews, the data collection seemed to be complete and appropriate for the purpose of answering our research question.

One of the most basic and important decisions in the content analysis was the selection of the meaning units and the further analysis into categories and themes, which was guided by the research question of the study. In this study the coding procedure was conducted to include those parts of the interview where the women talked about how they experienced working life and their positions, and how their family and social life was linked to this from a health and work (in)ability perspective. The credibility was strengthened through discussions between the co-researchers. Regarding transferability of the findings, we suggest that they might be transferred to similar settings and contexts: women working in high-level positions in the Swedish private sector, from similar socio-economic positions, and with similar demographics.

## Conclusions

Our results should be interpreted in the context of the present situation for white-collar women in high-level positions in male-dominated areas of working life in western countries. The findings indicate that there are factors in working life, as well as in private life, which play an important role in the informants' deteriorated health and lead to long-term sick leave. Job and workplace mismatching, as well as restructuring and profitability problems in companies probably lead to shortcomings in leadership performance, and possibly also to sex discrimination. Among the women in this study all these factors contributed to stress-related ill health and burnout syndromes, resulting in reduced working capacity and later sick leave. The informants were probably not able to cope well enough to maintain their health and well-being. However, on the basis of these findings, it should be possible to pay more attention to early indicators of exhaustion and implement measures at an early stage, in order to promote retained work ability by developing work policies and work settings which are adjusted to both men and women in management positions. One of the implications is that work mobility should be facilitated to match competence and interest. Social support at work, including leadership, should also be promoted, considering that not all members of management teams "...always have a wife".

## Competing interests

The authors declare that they have no competing interests.

## Authors' contributions

HS and MR participated in the design, data collection and analysis. HS was the main author of the manuscript. All authors have read and approved the final manuscript.

## Pre-publication history

The pre-publication history for this paper can be accessed here:

http://www.biomedcentral.com/1471-2458/10/210/prepub

## References

[B1] Helfat CE Harris D Wolfson PJ The pipeline to the top: women and men in the top executive ranks of U.S. corporations Academy of Management Perspectives 2006 20 42 64

[B2] Heilman ME Description and prescription: How gender stereotypes prevent women's ascent up the organizational ladder Journal of Social Issues 2001 57 657 74 10.1111/0022-4537.00234

[B3] OECD (2002-2004), Babies and Bosses - Reconciling Work and Family Life, series, OECD http://www.oecd.org/document/35/0,3343,en_2649_34819_34905443_1_1_1_1,00.html

[B4] Education at a Glance: OECD Indicators, OECD, Paris 2006

[B5] Marklund S Palmer E Hogstedt C Bjurvald M Theorell T Den höga sjukfrånvaron - sanning och konsekvens [High rates of sickness absence - truth and consequence] 2004 Swedish National Institute of Public Health, Stockholm 11 14

[B6] Försäkringskassan [The Swedish Social Insurance Agency]. Social insurance statistics http://statistik.forsakringskassan.se/portal/page?_pageid=93,237558&_dad=portal&_schema=PORTAL

[B7] Alecta http://www.alecta.se/templates/Sida____1607.aspx

[B8] Bäckman O Edling C Marklund S Work Environment and Work-related Health Problems in the 1990s 2000 Stockholm, Worklife and Health in Sweden 101 117 [National Institute for Working Life and Swedish Work Environment Authority.]

[B9] Bergendorff S Cohen Birman M Eklund M Gardberg Morner C Lidwall U Olsson S Andersson BM Women, Men and Sickness Absence Social Insurance in Sweden 2004 2004 Stockholm: The National Social Insurance Board 1 116

[B10] Vahtera J Kivimäki M Pentti J Linna A Virtanen M Virtanen P Ferrie JE Organisational downsizing, sickness absence, and mortality: 10-town prospective cohort study BMJ 2004 328 555 9 10.1136/bmj.37972.496262.0D 14980982 PMC381046

[B11] Sparks K Faragher B Cooper CL Well-being and occupational health in the 21st century workplace Journal of Occupational Organizational Psychology 2001 74 489 509 10.1348/096317901167497

[B12] Westerlund H Ferrie J Hagberg J Jeding K Oxenstierna O Theorell T Workplace expansion, long-term sickness absence, and hospital admission Lancet 2004 363 1193 7 10.1016/S0140-6736(04)15949-7 15081652

[B13] Ilmarinen J Tuomi K Klockars M Changes in the work ability of active employees over an 11-year period Scand J Work Environ Health 1997 23 suppl 1 49 57 9247995

[B14] Polit DF Tatano Beck CT Nursing Research. Principles and Methods 2006 Philadelphia: Lippincott Williams & Wilkins

[B15] Kvale S Interviews: an introduction to qualitative research interviewing 1996 Thousand Oaks, California: Sage

[B16] Mishler EG Research interviewing. Context and Narrative 1986 Cambridge: Harvard University Press, Ma

[B17] Försäkringskassan [The Swedish Social Insurance Agency]. Social insurance statistics http://www.forsakringskassan.se/nav/5b2a0fd19297a529864dad531a4d3414

[B18] Alecta http://www.alecta.se/templates/Sida____1607.aspx

[B19] Silverman D Interpreting Qualitative Data Method for Analysing Talk, Text and Interaction 2001 London: Sage

[B20] Tesch R Qualitative research. Analysis types and software tools 1991 New York:The Falmer Press

[B21] Crabtree BF Miller WL Doing Qualitative Research 1992 London: Sage Publications

[B22] Kondracki NL NS Wellman NS Amundson DR Content Analysis: Review of Methods and Their Applications in Nutrition Education Journal Nutrition Education Behaviour 2002 34 224 30 10.1016/S1499-4046(06)60097-3 12217266

[B23] Krippendorff K Content analysis: an introduction to its methodology 2004 Beverly Hills, Calif: Sage

[B24] Miller Franco I Bennet S Kanfer R Health sector reform and public health worker motivation: a conceptual framework Social Science & Medicine 2002 54 1255 66 10.1016/s0277-9536(01)00094-6 11989961

[B25] Tuomi K Ilmarinen J Jahkola A Katajarinnem L Tulkki A Work Ability Index 1998 Helsinki: Finnish Institute of Occupational Health

[B26] Nygard CH Arola H Siukola A Savinainen M Luukkaala T Taskinen H Virtanen P Perceived work ability and certified sickness absence among workers in a food industry Int Congr Ser 2005 1280 296 300 doi:10.1016/j.ics.2005.02.062 10.1016/j.ics.2005.02.062

[B27] Reiso H Nygard JF Brage S Gulbrandsen P Tellnes G Work ability and duration of certified sickness absence Scandinavian Journal of Public Health 2001 29 218 25 doi:10.1177/14034948010290031201 10.1177/14034948010290031201 11680774

[B28] Östlin PO Wamala SP, Lynch J Gender inequalities in health: the significance of work, in: Gender and Social Inequities in Health A Public Health Issue 2002 Lund: Studentlitteratur 43 65

[B29] Nermo M Structured by Gender. Patterns of sex segregation in the Swedish labour market. Historical and cross-national comparisons PhD thesis 1999 Swedish Institute for Social Research, no 41, Stockholm

[B30] Kivimäki M Feldt J Vahtera J Nurmi JE Sense of coherence and health: evidence from two cross-lagged longitudinal samples Social Science & Medicine 2000 50 583 97 10.1016/s0277-9536(99)00326-3 10641809

[B31] Moss Kanter R Men and women of the corporation 1977 New York: Basic Books

[B32] Heilman ME Sex bias in work settings: The lack of fit model Research in Organizational Behavior 1983 5 269 298

[B33] Heilman ME Description and prescription: How gender stereotypes prevent women's ascent up the organizational ladder Journal of Social Issues 2001 57 657 74 10.1111/0022-4537.00234

[B34] Lyness KS Thompson DE Climbing the Corporate Ladder: Do Female and Male Executives Follow the Same Route? Journal of Applied Psychology 2000 85 86 101 10.1037/0021-9010.85.1.86 10740959

[B35] Heilman MA Wallen D Fuchs M Tamkins Penalties for success. Reactions to women who succeed at male gender-typed tasks Journal of Applied Psychology 2004 89 416 27 10.1037/0021-9010.89.3.416 15161402

[B36] Fahlén G Goine H Edlund C Arrelöv B Knutsson A Peter R Effort-reward imbalance, "locked in" at work, and long-term sick leave International Archives of Occupational and Environmental Health 2009 82 191 7 DOI 10.1007/s00420-008-0321-5 10.1007/s00420-008-0321-5 18418625

[B37] Siegrist J Adverse health effects of high effort--low reward conditions at work Journal of Occupational Health Psychology 1996 1 27 43 10.1037/1076-8998.1.1.27 9547031

[B38] Siegrist J Social reciprocity and health: new scientific evidence and policy implications Psychoneuroendocrinology 2005 30 1033 38 10.1016/j.psyneuen.2005.03.017 15951124

[B39] Maslach C Schaufeli WB Leiter MP Job burnout Annual Review of Psychology 2001 52 397 422 10.1146/annurev.psych.52.1.397 11148311

[B40] Hallsten L Josephson M Torgén M Performance-based self-esteem: A driving force in burnout processes and its assessment 2005 Arbete och Hälsa (Work and Health), Stockholm:Sweden; National Institute for Working Life 4

[B41] Karasek RA Theorell T Healthy Work: Stress, Productivity, and the Reconstruction of Working Life 1990 New York: Basic Books

[B42] Johnson JV Hall EM Theorell T Combined effects of job strain and social isolation on cardiovascular disease morbidity and mortality in a random sample of the Swedish male working population Scandinavian Journal of Work, Environment and Health 1989 15 271 9 10.5271/sjweh.1852 2772582

[B43] Stanfeld S Candy B Psychosocial work environment and mental health - a meta-analytic review Scandinavian Journal of Work, Environment and Health 2006 32 443 62 10.5271/sjweh.1050 17173201

[B44] Borritz M Bültmann U Rugulies R Christensen KB Villadsen E Kristensen TS Psychosocial work characteristics as predictors for burnout: findings from a 3-year follow-up of the PUMA Study Journal of Occupational Environmental Medicine 2005 47 1015 25 10.1097/01.jom.0000175155.50789.98 16217242

[B45] Magnusson Hanson LL Theorell T Oxenstierna G Hyde M Westerlund H Demand, control and social climate as predictors of emotional exhaustion symptoms in working Swedish men and women Scandinavian Journal of Public Health 2008 36 737 43 doi:10.1177/1403494808090164 10.1177/1403494808090164 18684778

[B46] North FM Syme S L Feeney A Shipley M Marmot M Psychosocial work environment and sickness absence among British civil servants: the Whitehall II study American Journal of Public Health 1996 86 332 40 10.2105/AJPH.86.3.332 8604757 PMC1380513

[B47] Lidwall U Marklund S What is healthy work for women and men? - A case-control study of gender- and sector-specific effects of psycho-social working conditions on long-term sickness absence Work 2006 27 153 63 16971762

[B48] Oxenstierna G Ferrie J Hyde M Westerlund H Theorell T Dual source support and control at work in relation to poor health Scandinavian Journal of Public Health 2005 33 455 63 10.1080/14034940510006030 16332610

[B49] Väänänen A Toppinen-Tanner S Kalimo R Mutanen P Vahtera J Peiró JM Job characteristics, physical and psychological symptoms, and social support as antecedents of sickness absence among men and women in the private industrial sector Social Science & Medicine 2003 57 807 24 10.1016/s0277-9536(02)00450-1 12850108

[B50] Lindell MK Brandt CJ Climate quality and climate consensus as mediators of the relationship between organizational antecedents and outcomes Journal of Applied Psychology 2000 84 640 47 10.1037/0021-9010.84.4.640 10900809

[B51] Hyde M Jappinen P Theorell T Oxenstierna G Workplace conflict resolution and the health of employees in the Swedish and Finnish units of an industrial company Social Science & Medicine 2006 63 2218 27 10.1016/j.socscimed.2006.05.002 16782255

[B52] Nyberg A Westerlund H Magnusson Hansson LL Theorell T Managerial leadership is associated with self-reported sickness absence and sickness presenteeism among Swedish men and women Scandinavian Journal of Public Health 2008 36 803 11 10.1177/1403494808093329 19004898

[B53] Lincoln YS Guba EG Naturalistic Inquiry 1985 Newbury Park, London; New Delhi: Sage Publications Inc

